# Colonic metastasis from breast carcinoma: A case report and systematic review of a rare clinical scenario

**DOI:** 10.1007/s00384-026-05102-0

**Published:** 2026-02-07

**Authors:** Matteo Matteucci, Gisella Barone, Lorenza Zampino, Carla Codecà, Vincenza Paola Dinuzzi, Aurora Battista, Umberto Rivolta, MiMi Yen, Marco Galliano, Camillo Leonardo Bertoglio

**Affiliations:** 1 Residency in General Surgery, University of Milan, Milan, Italy; 2 Department of General Surgery, Ospedale G. Fornaroli, ASST-Ovest Milanese, Magenta, Italy; 3 Department of Oncology, Ospedale G. Fornaroli, ASST-Ovest Milanese, Magenta, Italy

**Keywords:** Breast cancer, Colon metastasis, Metastatic breast tumor, Case report, Hemicolectomy

## Abstract

**Purpose:**

Colonic metastasis from breast cancer is extremely rare, with an incidence of only 0.1%. Diagnosis is often difficult and guidelines are not yet established. The aim of our review is investigating the latency from the primary tumor, the common symptoms, the diagnostic and therapeutic strategies and the role of surgery for this rare clinical scenario.

**Materials:**

We report the case of a 57-year-old woman with multiple colonic metastasis from primary breast tumor, who underwent laparoscopic left hemicolectomy. A systematic review of 64 case reports was also conducted.

**Results:**

Lobular carcinoma is more frequently associated with gastro-intestinal (GI) metastasis than ductal carcinoma. The median age at diagnosis is 65.5 (IQR = 15) years with colonic metastases typically occurring after a median of 8 years (IQR = 13) from the primary tumor diagnosis. The most frequent symptoms are abdominal pain (34.4%), bowel habit changes (26.6%), and intestinal obstruction (9.4%). In 25% of cases, metastases were incidentally discovered during follow-up. The median disease-free survival was 12 months (IQR = 27.5). Thirteen studies reported death at a median of 12 months (IQR = 20), while 24 did not report follow-up data.

**Conclusions:**

The poor prognosis is mainly due to long latency between primary diagnosis and metastasis onset, as well as to non-specific symptoms. Immuno-histochemical is crucial for diagnosis, although not sufficient to determine tumor origin definitively. Patients with history of breast cancer presenting with GI symptoms should undergo prompt endoscopic evaluation, although routine surveillance remains controversial. Surgery may be considered in selected cases, but systemic therapies remain the cornerstone of treatment.

**Supplementary Information:**

The online version contains supplementary material available at 10.1007/s00384-026-05102-0.

## Introduction

Breast cancer is the most common tumor in the female sex, with a worldwide increasing incidence. According to the more recent Italian Association of Medical Oncology (A.I.O.M.) guidelines, published in 2023, and to Italian Association Tumor Register (A.I.R.TUM.) data, in Italy breast cancer is the most commonly diagnosed tumor in women regardless of the age group, with an incidence of 41% in the age group 0–49 year-old (y.o.), 35% in the 50–69 y.o and 22% in women with an age superior of 70 y.o. [1]. It represents the first world cause of women death due to tumor disease and in Italy in 2020, 12,300 deaths due to breast cancer were estimated [1]. On a global scale, the burden is similarly substantial: in 2022, an estimated 2.3 million new cases of female breast cancer were diagnosed worldwide, making it the most diagnosed cancer in women in 157 of 185 countries. At the same time, breast cancer caused approximately 670,000 deaths in that year [2]. The Global Cancer Observatory (GLOBOCAN) (2022) [3] reports that breast cancer accounted for approximately 46.8 new cases per 100,000 women globally, representing the second most frequent cancer overall across both sexes. These data highlight both the high incidence and the rising trend of breast cancer globally, underscoring the imperative for early detection, effective treatment strategies and targeted prevention efforts.

Breast cancer generally metastasizes to the lungs, liver, bones, and brain and only rarely to the gastrointestinal (GI) tract. In this scenario, metastasis generally affect the stomach and small bowel, while colonic metastasis is extremely rare, with a worldwide incidence of 0.1% reported in literature [4].

We aim to describe the management of a patient with multiple colonic metastasis after previous breast cancer surgery and to perform a systematic review of the case reports available in literature.

Supplementary digital continent 1 (SDC1) reports the list of the abbreviations and their definitions used in the manuscript.

## Materials and methods

### Systematic review

Two authors (M.M. and G.B.) independently performed a case reports systematic review of colic metastasis from breast cancer on the Pubmed database and EMBASE-Medline, updated to 1 June 2025, with a return of 194 and 581 articles, respectively. In order to conduct the research, the following combination of keywords was employed: colon metastasis or colonic metastasis or gastrointestinal metastasis and breast cancer or breast tumor.

The inclusion criteria were case reports and case series written in English, with the full text available. Exclusion criteria included abstracts, congress publications and case reports involving patients with concomitant malignancies in addition to breast carcinoma.

Out of these, two authors (M.M. and G.B.) independently excluded those that were written in a non-English language, with 324 articles selected. After a screening of the titles and the abstract and the exclusion of article with no text available, a total of 64 articles were included in our review. The study was conducted in accordance with the PRISMA (Preferred Reporting Items for Systematic Reviews and Meta-Analyses) guidelines [5] (SDC2). The protocol for this study was registered on PROSPERO (CRD420251119141).

Our primary outcomes include the interval between the diagnosis of the primary tumor and the onset of metastases, the most frequently reported presenting symptoms, the diagnostic modalities employed, the therapeutic strategies adopted, and the potential role of surgical intervention.

This review is based solely on case reports and case series, which are subject to publication bias and heterogeneous reporting. These limitations reduce the generalizability and strength of the evidence and preclude formal meta-analysis. However, in the context of rare conditions, such as colon metastasis from breast cancer, this approach remains useful for highlighting clinical patterns and management strategies. A more detailed discussion of these limitations is provided later in the manuscript.

### Assessment of risk of bias in included studies

For each included study, two reviewers (M.M., G.B.) independently assessed the risk of bias. Disagreements were resolved by consensus; if consensus could not be reached, a third reviewer (L.Z.) was consulted. The review team then reached a decision by consensus. We used the Joanne Briggs Institute (JBI) critical appraisal tools [4], useful for classifying the risk of bias into four categories (yes, no, unclear, not applicable).

## Results

### Case report

A 57-year-old woman underwent breast cancer surgery including quadrantectomy of the left breast and left axillary lymph node dissection, due to a positive sentinel lymph node biopsy. She was diagnosed with estrogen receptor (ER) positive, progesterone receptor (PgR) positive, human epidermal growth factor receptor 2 (HER2/neu) negative, pT1c N1a invasive lobular carcinoma (ILC). She received radiotherapy and began hormonal therapy with anastrozole from October 2018, since November 2023. Follow-up was carried out regularly, with no evidence of disease recurrence.

**Fig. 1:**
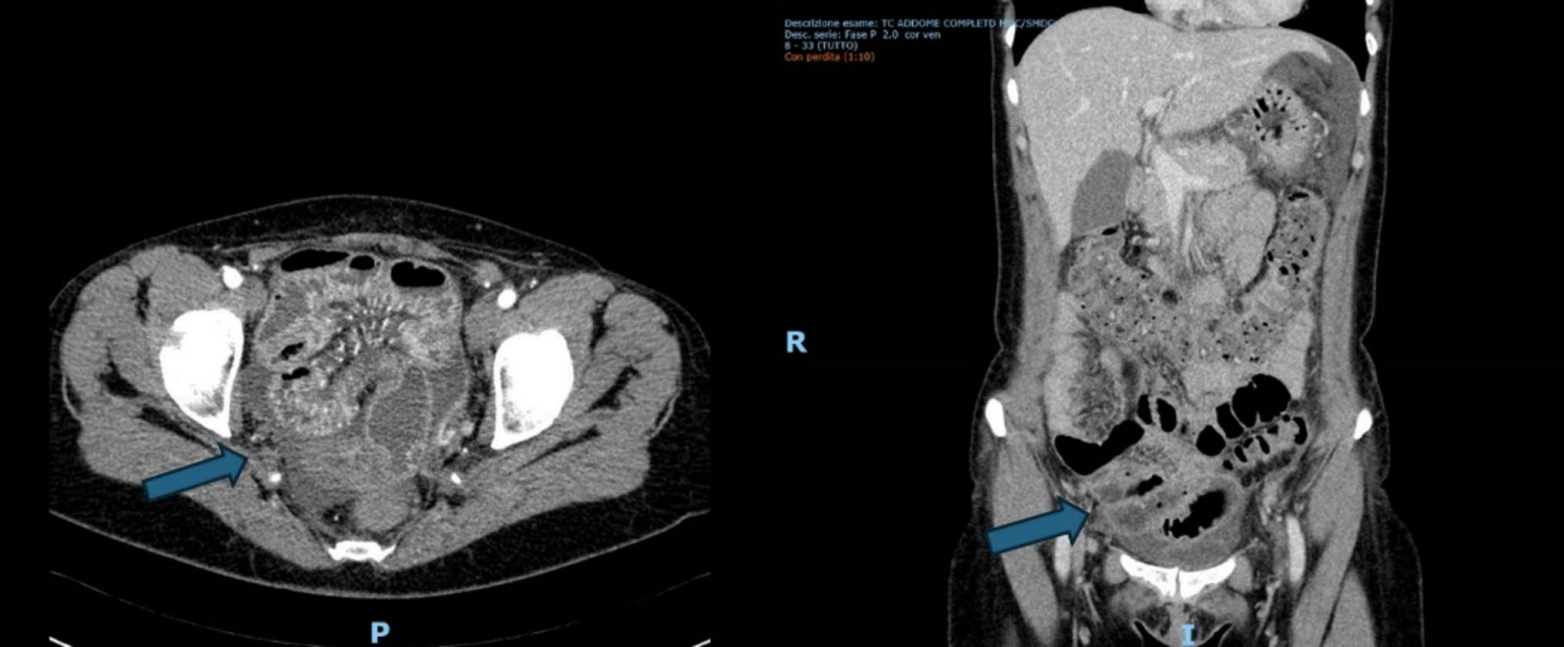
Emergency abdominal CT scan. The arrows showed the “*Distension of the small bowel loops in the pelvis with 15 cm slightly thickened—edematous and hypovascularized small bowel walls as in ischemic ileitis (post-embolic?), in the absence of significant alterations in the canalization of the main tributary vessels”*

In November 2023, she was admitted to our emergency department with intermittent abdominal pain. An emergency abdominal CT scan revealed a small bowel obstruction, without any colonic pathological findings, that was successfully treated with a conservative approach (Fig. 1).

**Fig. 2:**
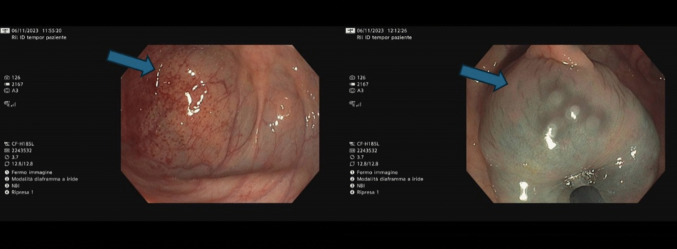
Colonoscopy. The arrows show sub-stenotic masses at level of descending colon and sigmoid colon

The patient subsequently underwent colonoscopy in December 2023 due to recurrent sub-occlusive episodes, leading to repeated emergency department admissions. The examination revealed three sub-stenotic masses located in the descending and sigmoid colon (Fig. 2). Biopsy results confirmed metastases from ILC. Immunohistochemistry showed ER positivity and PgR negativity. HER2/Neu overexpression was negative and the proliferation index (MIB-1) was 8–10%.

A PET/CT scan was performed in December 2023, confirming the presence of colonic metastases without evidence of further disease dissemination. The case was discussed by a multidisciplinary oncologic team. Based on the extent and biological characteristics of the disease, systemic treatment with Fulvestrant and Palbociclib was initiated in December 2023. A follow-up PET/CT scan demonstrated oncologic stability, and the therapeutic regimen was continued.

**Fig. 3:**
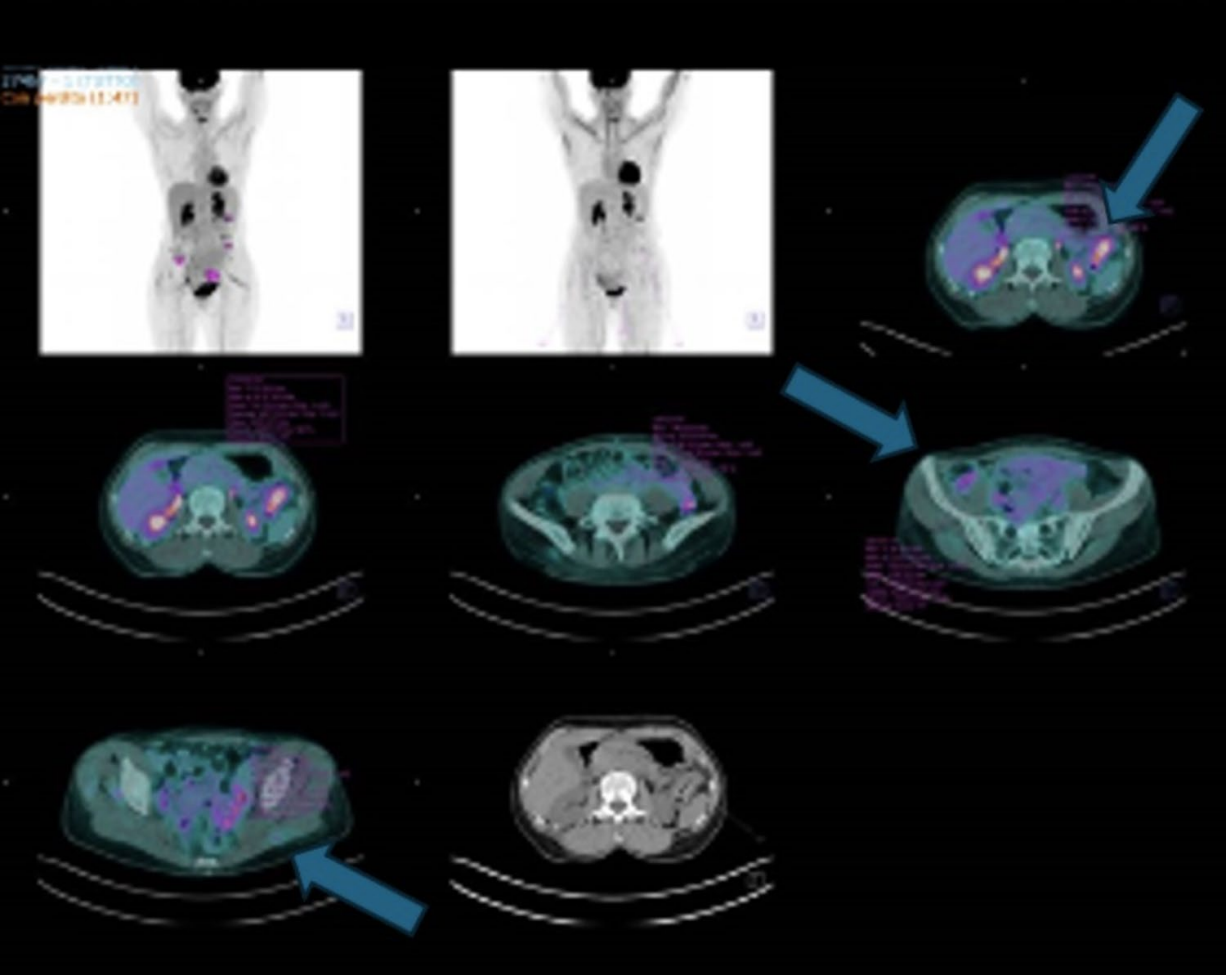
PET-CT scan performed in December 2024. The arrows show *“Increase in both the extent and intensity of radiopharmaceutical uptake of the known left colic lesion just inferior to the splenic flexure (SUV 7,73). Appearance of glucose hypermetabolism in the colon in the bilateral iliac fossa and at the level of the sigmoid colon. These findings are suspicious for localization of metabolically active secondary disease”*

However, in December 2024, a subsequent PET/CT scan revealed disease progression, with an increase in both the extent and intensity of metabolic uptake (SUV max 7.73 vs. 4.94). Additionally, new areas of increased metabolic activity were identified in the right and sigmoid colon (Fig. 3).

**Fig. 4:**
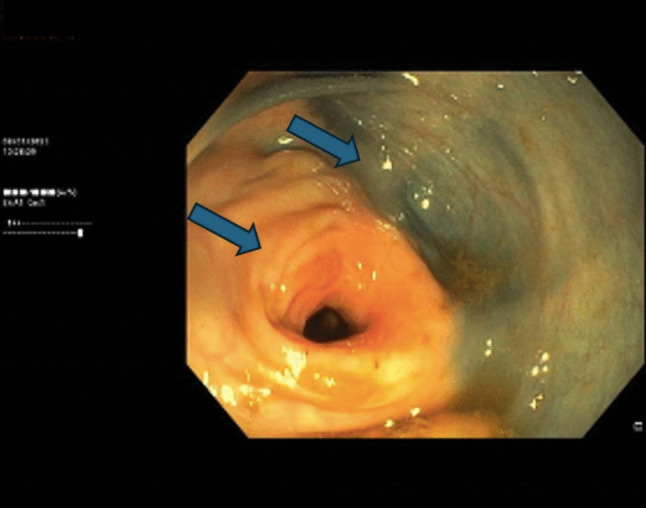
Colonoscopy with the arrows showing one of the sub-stenotic lesions in the descending colon

Colonoscopy performed in December 2024 revealed two cancer-related sub-stenoses located 27 and 36 cm from the anal verge, which prevented completion of the endoscopic examination (Fig. 4). Biopsies were taken and histological analysis confirmed colonic metastases from ILC.

**Fig. 5:**
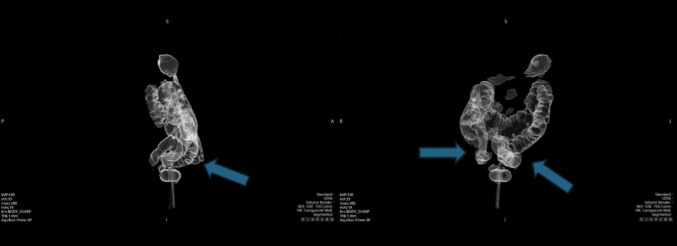
Colon-CT scan. Arrows show *“A stenosing parietal lesion is recognized at the recto-sigmoid junction. A stenosing lesion is recognized at the level of the distal descending colon, extending for a distance of approximately 2 cm. A larger stenosing lesion is recognized along the proximal third of the descending colon, extending for approximately 4 cm. No pathological lesions are recognized in the transverse colon or ascending colon”*

A subsequent CT colonography in January 2025 confirmed the presence of the previously described lesions but did not show any abnormalities in the right colon, contrary to the PET/CT findings (Fig. 5).

**Fig. 6:**
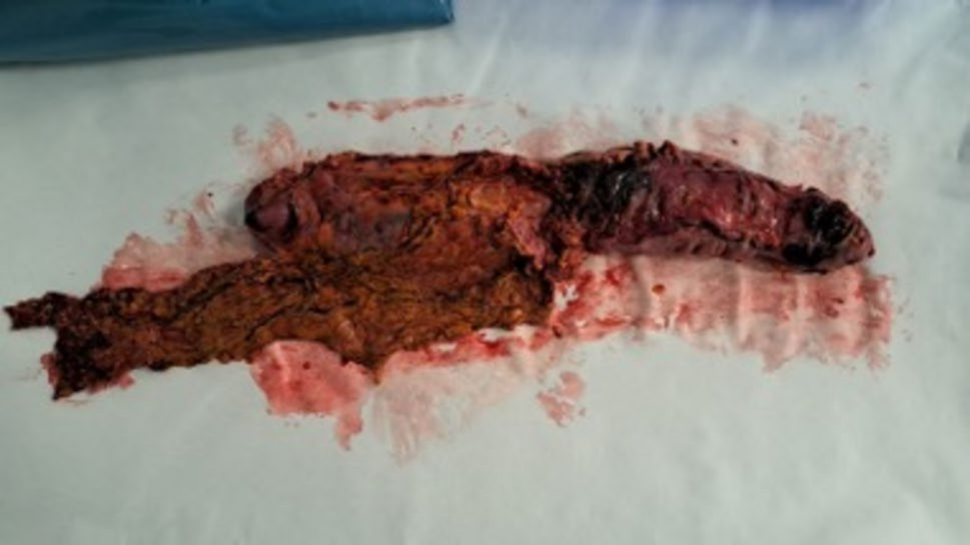
The photo shows the operative field with the metastatic lesion at level of descending colon and rectum-sigmoid junction

The patient underwent laparoscopic left colectomy in February 2025 (Fig. 6). The procedure, performed under general anesthesia, lasted approximately 4 h. Postoperative recovery was complicated by a delayed ileus due to an internal hernia, which required surgical reintervention. The patient was discharged 7 days after the initial surgery.

**Fig. 7:**
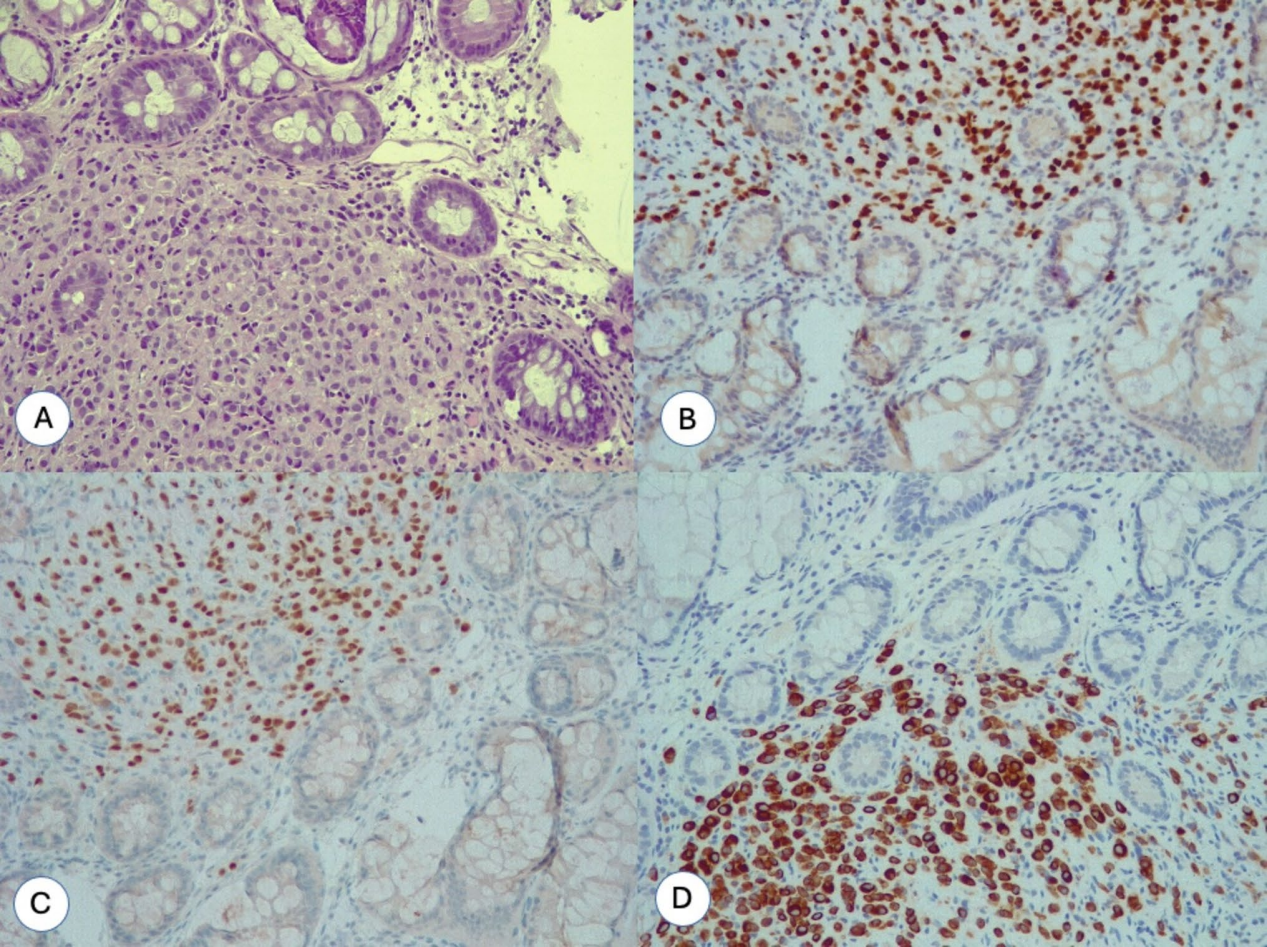
**A** The photo shows the lamina propria of the intestinal mucosa infiltrated by monomorphic neoplastic cells of intermediate size (hematoxylin eosin, 20 ×). **B** The neoplastic cells that infiltrate the lamina propria of the intestinal mucosa are of epithelial origin and are immunoreactive for cytokeratin 7 (CK7, 20 ×). **C**–**D** Neoplastic epithelial cells are immunoreactive for GATA3 and estrogen receptors. These two markers confirm the mammary origin of the metastasis

Histological examination confirmed multiple colonic metastasis from primary breast cancer with peri-visceral lymph-nodes involvement. Immunohistochemistry revealed ER-positive, PgR-positive, and HER2/Neu negative status (Fig. 7). The patients started the oncologic follow-up and chemotherapy treatment with oral capecitabine was prescribed.

The patient remains under regular oncological surveillance. At six months following the commencement of capecitabine therapy, follow-up evaluations have demonstrated no evidence of disease recurrence or progression to date.

The patient is receiving systemic treatment with capecitabine and at 8-month postoperative follow-up, there is no clinical or radiological evidence of disease recurrence.

Written informed consent was obtained from the patient for the publication of this case report and accompanying images and the investigations were conducted in accordance with the principles outlined in the Declaration of Helsinki. The case report was conducted in accordance with the CARE (CAse REport) guidelines [6] (SDC3).

### Results of systematic review

**Fig. 8:**
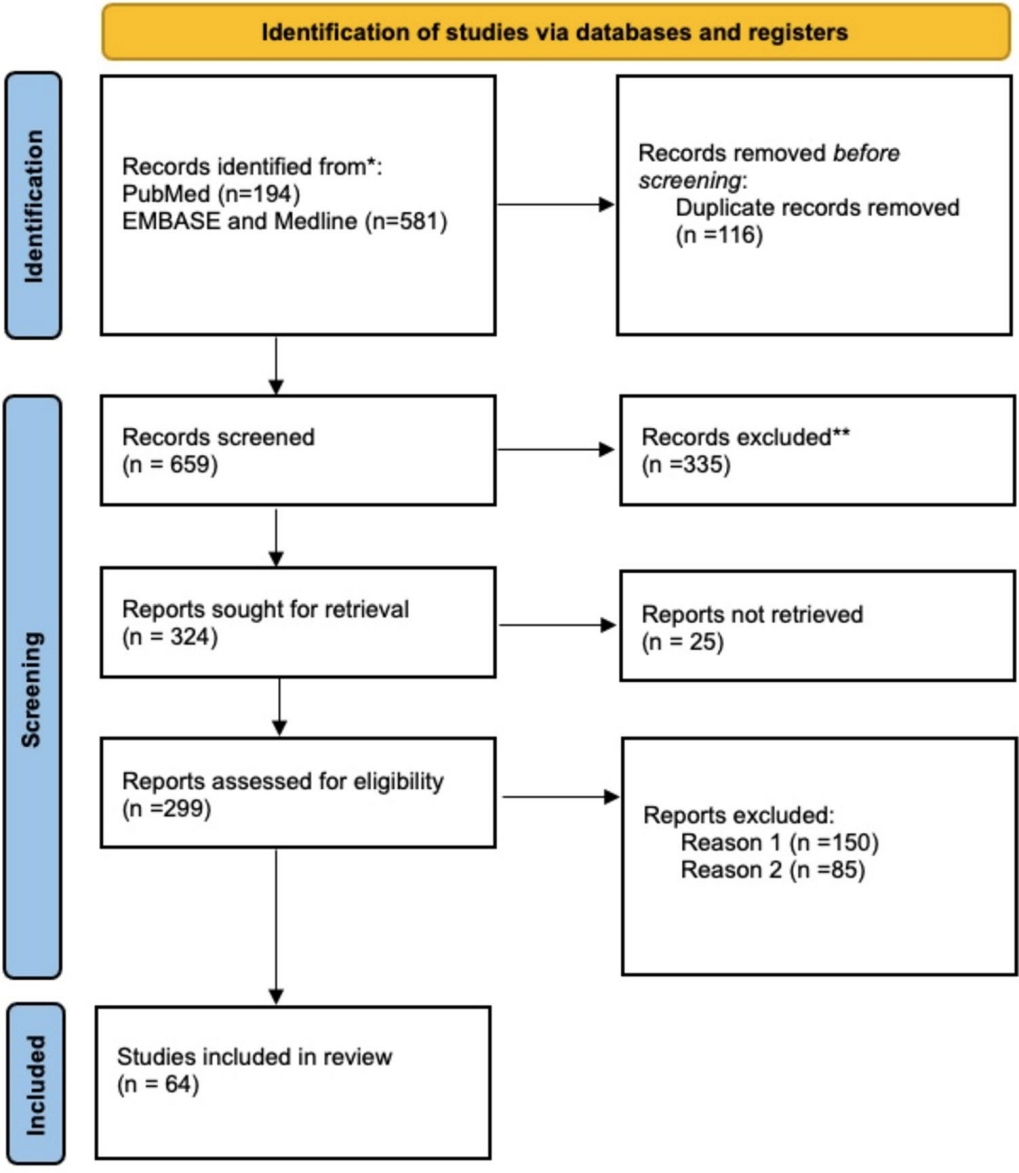
The PRISMA flowchart of included studies. Records excluded**: exclusion of non-English written case reports; records excluded Reason 1: exclusion of abstract or congress publications; records excluded Reason 2: exclusion of case reports involving patients with concomitant malignancies in addition to breast carcinoma

A total of 64 case-reports [7–70] were included in our literature review. The PRISMA flow diagram for study selection is shown in Fig. 8.

For each study, two authors (M.M. and G.B.) independently extracted the following data: patient age, sex, site, and histology of the primary breast tumor, biomarker status (when available) of the primary breast tumor and colonic metastases, treatment modalities (surgery, chemotherapy, hormonal therapy, or radiotherapy), site of metastases and the time interval between the diagnosis of the primary tumor and the onset of metastatic disease. The characteristics of patients and studies included are reported in Table 1.

**Table 1:** Characteristics of case reports included in our review

Author	Age	Site primary tumor	Pathological types of primary tumor	Biomarkers	Treatment	Time since first diagnosis (years)	Site of metastasis	Biomarkers
Tekeuchi [7]	38	Left	ILC	ER +, PgR- HER2/Neu-	Surgery, CT, RT, Endocrine Therapy	3	Stomach and Colon	ER +, PgR-, HER2/Neu-
Jansesn van Rensburg [8]	74	Left	DC	ER +, PgR -	Surgery, RT, Endocrine Therapy	27	Bone, Ovary and sigmoid colon	ER +, PgR +, E-cadherin +, GATA 3 +, CK20-
Higley [9]	74	Right	-	-	Surgery, RT, Endocrine Therapy	40	Transverse Colon	ER-, PgR-, HER2/Neu-, E-cadherin -, GATA 3 +, CK20 -
Khan [10]	56	Right	Ring cell adenocarcinoma	-	-	Simultaneous	Stomach, small bowel and colon	-
Noor [11]	68	-	ILC	ER +, PgR + HER2/Neu-	Surgery, RT, Endocrine Therapy	30	Bone and sigmoid colon	ER-, PgR +, HER2/Neu-
Bering [12]	67	Left	ILC	ER +, PgR + HER2/Neu-	Endocrine Therapy	Simultaneous	Transverse colon	ER-, PgR- HER2/Neu +, GATA3 +, CK20-, E-cadherin-
Abid [13]	59	Left	LC	ER +, PgR + HER2/Neu-	-	Simultaneous	Stomach, Duodenum and colon	ER +, PgR +
Takedomi [14]	76	Right	LC	-	CT	Simultaneous	Descending colon	-
Abu Zaanona [15]	73	-	-	-	Endocrine Therapy	-	Colon	ER +, PgR- HER2/Neu-, GATA3 +, E-cadherin
Inoue [16]	63	Right	-	-	CT, Endocrine Therapy	15	Colon	ER +, PgR- HER2/Neu-, GATA3 +, CK20-, E-cadherin
Mostafa [68]	56	-	IDC	ER +, PgR +	Surgery, RT, Endocrine Therapy	5	Descending colon	ER-, PgR-
Tsujimura [17]	51	Left	ILC	ER +, PgR +	-	Simultaneous	Ileocecal	ER +, PgR +, HER2/Neu-
Feng [18]	49	Right	IDC	-	-	2	Colon	-
Kobayashi [19]	74	-	ILC	-	Surgery	23	Stomach and Colon	ER +, E-cadherin-
Théraux [67]	69	Bilateral	IDC	-	Surgery, Endocrine Therapy	28	Transverse Colon	ER +, PgR +, HER2/Neu-, CK20-
Malhotra [24]	71	-	-	-	-	-	Bone and Transverse Colon	-
Wang [25]	70	Left	IDC	E-cadherin +, CK20 +	Surgery, RT, Endocrine Therapy	10	Ascending Colon	ER +, CK20 +, E-cadherin +
Algethami [26]	47	Bilateral	ILC	ER +, PgR + HER2/Neu-	Surgery, RT, Endocrine Therapy	4	Rectum	ER +. HER2/Neu +
Zhou [27]	54	Right	IDC	ER +, PgR + HER2/Neu-	Surgery, RT, CT, Endocrine Therapy	8	Sigmoid colon	ER-, PgR- HER2/Neu-, CK20-
Andriola [28]	63	Left	IDC-LC	-	Surgery, RT, CT, Endocrine Therapy	23	Colon and Terminal ileum	HER2/Neu +
Motos-Mico [29]	69	Right	ILC	-	Surgery, CT	18	Sigmoid Colon	ER-, PgR- HER2/Neu-,
Abdallah [30]	66	Right	ILC	ER +, PgR + HER2/Neu-	-	Simultaneous	Colon	GATA3 +, ER +, CK20-
Michalopoulos [31]	55	Left	IDC	-	Surgery, CT	4	Transverse Colon	-
Michalopoulos [31]	57	Left	ILC	-	Surgery, CT, RT	10	Transverse Colon	-
Schellenberg [32]	69	Left	IDC	ER +, PgR + HER2/Neu +, E-cadherin +	Surgery, RT, CT, Endocrine Therapy	2	Rettosigmoid	ER +, PgR +, E-cadherin +, CK20-
Matsuda [33]	62	Left	ILC	-	Surgery	24	Ascending colon and Rectum	ER +, CK20- E-cadherin -
Koleilat [34]	54	Right	IDC	-	Surgery, RT, CT, Endocrine Therapy	13	Colon	ER +, PgR + HER2/Neu-
Mistrangelo [35]	80	Left	ILC	-	Surgery, Endocrine Therapy	25	Sigmoid Colon	-
Razzetta [36]	77	Bilateral	ILC (left), IDC (right)	-	-	Simultaneous	Right, transverse and left colon	ER +, PgR-
Cervi [37]	59	-	ILC	-	Surgery	8	Rectum	ER +, PgR-
Amin [38]	61	Right	ILC	-	Surgery, Endocrine Therapy	17	Rectum	ER +, CK7 +, PgR +, CK20-
Lopez Deogracias [39]	67	Left	ILC	ER +, PgR +	Surgery	15	Rectum	ER-, PgR-, CK20-
Law [40]	49	Left	IDC	ER +	Surgery, Endocrine Therapy	5	Descending Colon	ER +
Samra[41]	64	-	-	-	-	-	Colon	CK7-, GATA3 +, ER +, CK20-, PgR-
Kim [23]	46	Right	Metaplastic	ER-, PgR-, HER2/Neu-	Surgery, CT, RT	2	Sigmoid Colon	CK20-, ER-, PgR-, HER2/Neu-, CK7 +
Signorelli [21]	62	Right	ILC	-	Surgery	12	Colon	ER +, PgR +, HER2/Neu-
Gizzi [42]	72	-	-	ER +, PgR +	Surgery, CT, RT, Endocrine Therapy	11	Sigmoid Colon	CK7 +, GATA3 +, ER +, PgR +, HER2/Neu-
Dhar [43]	75	Left	-	ER +	Surgery	6	Sigmoid Colon	ER +, CK7 +, CK20-
Maekawa [44]	52	Right	IDC	ER +, PgR +	Surgery, CT, RT, Endocrine Therapy	16	Ascending Colon	-
Koufopoulos [45]	87	-	-	-	-	-	Colonic serosa	CK20-, CK7-, GATA3 +, ER +, PgR +, E-cadherin -
Critchley [46]	62	-	IDC	-	Surgery, CT, RT, Endocrine Therapy	8	Stomach, Ascending Colon	ER +, PgR +, HER2/Neu-, CK7 +, CK20-
Kachi [47]	58	Left	ILC	-	Surgery, CT, RT. Endocrine Therapy	6	Sigmoid colon and Appendix	ER +, PgR +
Blachman-Braun [48]	73	Bilateral	-	-	Surgery, CT	15	Colon	ER +, PgR-, HER2/Neu-
Jia [21]	67	Right	IDC	ER +, PgR +, HER2/Neu-	Surgery, CT, Endocrine Therapy	10	Appendix, Colon	GATA3 +, ER +, PgR-, HER2 +/Neu, E cadherin +, CK7 +, CK20-
Arif [20]	65	Bilateral	IDC	Triple negative (left), HER2/Neu + (right)	-	-	Descending colon	
Katz [49]	68	Left	IDC	ER +, PgR +, HER2/Neu +	Surgery, CT, RT, Endocrine Therapy	-	Ascending colon and appendix	CK7 +, ER +, GATA3 +, CK20-, E-cadherin +
Falco [50]	67	Left	ILC	ER +, PgR +, HER2/Neu-	Surgery, CT, Endocrine Therapy	14	Ileocecal Valve	ER +, PgR +, HER2/Neu-
Do [51]	67	Right	ILC	-	Surgery, CT, Endocrine Therapy	7	Ascending Colon	GATA3 +
Jones [70]	55	Right	IDC	ER +, PgR +, HER2/Neu-	Endocrine Therapy	-	Cecum, Appendix	GATA3 +, CDX-
Imai [51]	72	Right	ILC	-	CT	2	Ascending Colon	CK20-, CK7 +, E-cadherin -, ER +
Nikkar.Esfahani [64]	63	Right	ILC	-	Surgery, Endocrine Therapy	17	Rectum	ER +, PgR-
Ikeda [62]	50	Left	-	-	Surgery, CT, RT	-	Sigmoid colon	
Okamura [63]	75	Bilateral	-	-	Surgery	-	Ascending Colon	CK7 +, CK20 +, ER +, PgR-, HER2/Neu +
Schwarz [53]	78	-	ILC/IDC	ER-/PgR-	-	0.25	Colon	-
Ambroggi [54]	40	Left	IDC	ER +, PgR +, HER2/Neu-	CT, RT, Endocrine Therapy	Simultaneous	Rectum	ER +, PgR +, HER2/Neu-
Lau [55]	61	Right	LC	-	Surgery, RT, Endocrine Therapy	5	Rectum	ER +, PgR +, HER2/Neu-
Santini [56]	78	Left	LC	ER +; PgR +	Surgery, CT	5	Ileocecal valve	ER +, PgR-; CK7 +
Laoutliev [57]	57	Right	LC	ER +	Surgery, RT, Endocrine Therapy	-	Rectum	ER +
Abdalla [58]	82	Right	ILC	ER +; PgR +	Endocrine Therapy	19	Ileocecal valve	ER +; CK7 +; CK20-; E-cadherin -
Bamias [59]	74	Right	-	ER-; PgR-	Surgery, CT, RT	10	Rectum	ER-; PgR-
Black [60]	66	Right	LC	ER +; PgR-; HER2/Neu-	-	6 months	Ascending Colon	CK7 +; CK20 +; ER +; PgR-; HER2/Neu-; GATA 3 +
Ng CE [61]	56	Right	ILC/DC	ER +; HER2/Neu-	CT	5	Rectum	ER +; CK7 +; HER2/Neu-; CK20-
Osaku [65]	69	Right	LC	-	CT Endocrine Therapy	Simultaneous	Rectum	
Rajan [66]	62	Left	IDC	ER +	RT, Surgery	2	Rectum	CK7 +, ER +, PgR-, HER2/Neu-
Li [69]	65	Left	IDC	ER +, PgR +, HER2/Neu +	CT	6	Ascending Colon	CK7 +, CK20-, GATA3 +, ER-, PgR-, HER2/Neu-
Our case report	57	Left	ILC	ER +, PgR +, HER2/Neu-	RT, Surgery Endocrine Therapy	5	Sigmoid colon Descending Colon	CK7 +, ER +, PgR +, HER2/Neu-, GATA 3 +

*ILC*, Invasive Lobular Carcinoma; *IDC*, Invasive Ductal Carcinoma; *DC*, Ductal Carcinoma; *LC*, Lobular Carcinoma; *ER*, estrogen receptor; *PgR*, Progesterone Receptor; *HER2/Neu*, Human Epidermal Growth Factor Receptor 2; *CT*, Chemotherapy; *RT*, Radiotherapy; *GATA3*, GATA Binding Protein 3; *CK7*, Cytokeratin 7; *CK20*, Cytokeratin 20; *CDX2*, Caudal-type Homeobox 2

In 21 case-reports, the primary breast tumor was an invasive lobular carcinoma (ILC), instead lobular carcinoma (LC) was described as primary breast tumor in 4 studies. In 18 studies patients were affected by invasive ductal carcinoma (IDC), while in one study a ductal carcinoma was reported. Both ILC and IDC were primary breast carcinoma in 4 studies. Khan et al. described one case of colonic metastasis from ring-cell adenocarcinoma, while in only one case report colonic metastasis originated from a metaplastic breast carcinoma. The remaining studies do not report the histopathology of primary breast tumor. All the studies reported metastatic breast women patients, except for one [68] that described a case of breast male patients with colonic metastasis. The median age at time of colonic metastasis was 65.5 (IQR = 15). The median time of metastasis’ diagnosis from first tumor diagnosis was 8 years (IQR = 13). 10 out of the articles reported a simultaneous diagnosis of colonic metastasis and primary breast tumor, while 10 of them do not report information regarding this topic.

Presentation symptoms at diagnosis, survival and time of death after diagnosis of the metastatic disease are reported in Table 2. In Table 2, clinical presentation categories are not mutually exclusive, as patients could present with multiple symptoms. Percentages indicate the proportion of patients exhibiting each symptom, and the denominators (n) specify the number of patients per category.

**Table 2:** Clinical features of case reports included in our review

Author	Clinical presentation or main symptom at diagnosis	Survival after the diagnosis of metastasis	Time of death after the diagnosis of metastasis
Tekeuchi [7]	Surveillance: screening colonoscopy	26 months	
Jansen van Rensburg [8]	Surveillance: screening colonoscopy	NED, no information about length of F.U	
Khan [10]	Bleeding, anemia	Death	No information
Higley [9]	Change of bowel habit	NED, no information about length of F.U	
Noor [11]	Nausea, Abdominal pain	Death	No information
Bering [12]	Change of bowel habit	2 months	
Abid [13]	Change of bowel habit, nausea	6 months	
Takedomi [14]	Surveillance: screening colonoscopy	No information	
Abu Zaanona [15]	Abdominal pain and change of bowel habit	Death	2 years
Inoue [16]	Surveillance: oncologic follow-up (PET-CT scan)	2 years	
Amin [38]	Change of bowel habit	No information	
Koleilat [34]	Abdominal pain	10 months	
Mistrangelo [35]	Bowel obstruction	Death	9 months
Matsudo [33]	Change of bowel habit	No information	
Maekawa [44]	Surveillance: oncologic follow-up (PET-CT scan and increase in CA 125)	Death	8 months
Nikkar-Estafhani [64]	Change of bowel habit	12 months	
Wang [36]	Abdominal pain	2 years	
Motos-Mico [29]	Unknown	12 months	
Andriola [28]	Unknown	No information	
Gizzi [42]	Abdominal pain	2 years	
Ikeda [62]	Abdominal pain	12 months	
Okamura [63]	Change of bowel habit	5 months	
Falco [50]	Surveillance: oncologic follow-up (PET-CT scan and increase in CA 125 and CEA)	4 months	
Katz [49]	Abdominal pain	No information	
Tsujimura [17]	Abdominal pain and change of bowel habit	9 months	
Feng [18]	Abdominal pain	No information	
Kobayashi [19]	Surveillance: screening colonoscopy	Death	15 months
Arif [20]	Bleeding	Death	1 months
Jia [21]	Abdominal pain	12 months	
Kim [22]	Surveillance: screening colonoscopy	No information	
Signorelli [23]	Bowel obstruction	20 months	
Malhotra [24]	Change of bowel habit	No information	
Algethami [26]	Abdominal pain	No information	
Zhou [27]	Abdominal pain	2 years	
Abdallah [30]	Abdominal pain	18 months	
Michalopoulos [31]	1. Abdominal pain, anemia 2. Bowel obstruction	1. Death 2. No information	1. 3 years
Schellenberg [32]	Bowel obstruction	Death	12 months
Razzetta [36]	Abdominal pain	No information	
Cervi [37]	Surveillance: screening colonoscopy	No information	
Lopez Deogracias [39]	Abdominal pain	Death	2 months
Law [40]	Abdominal pain	No information	
Samra [41]	Surveillance: screening colonoscopy	Death	18 months
Dhar [43]	Perforation	No information	
Koufopoulos [45]	Abdominal pain, bleeding	No information	
Critchley [46]	Anemia, change of bowel habit	2 months	
Kachi [47]	Change of bowel habit	5 years	
Blachman-Braun [48]	Bowel obstruction	No information	
Imai [50]	Surveillance: oncologic follow-up (CT scan)	No information	
Do [52]	Surveillance: screening colonoscopy	No information	
Schwarz [53]	Surveillance: screening colonoscopy	19 months	
Ambroggi [54]	Bleeding	NED, no information about length of F.U	
Lau [55]	Change of bowel habit	2 years	
Santini [56]	Surveillance: oncologic follow-up (increase in CA 19.9)	12 months	
Lautliev [57]	Change of bowel habit	No information	
Abdalla [58]	Abdominal pain, vomiting	No information	
Bamias [59]	Change of bowel habit	Death	6 months
Black [60]	Abdominal pain	No information	
NgCE [61]	Surveillance: screening colonoscopy	6 months	
Osaku [65]	Change of bowel habit	Death	4 years
Rajan [66]	Bleeding	5 years	
Theraux [67]	Bowel obstruction	12 months	
Mostafà [68]	Change of bowel habit	No information	
Li [69]	Bleeding	20 months	
Jones [70]	Abdominal pain, vomiting	12 months	

*NED*, no-evidence of disease; *F.U.*, follow-up

The most common presentation was abdominal pain (32.3%) (*n* = 21), followed by change of bowel habit (24.6%) (*n* = 16), bowel obstruction (9.2%) (*n* = 6), bleeding (7.7%) (*n* = 5) and perforation (1.5%) (*n* = 1). In 23% of cases (*n* = 15), the diagnosis of colonic metastasis was established during routine surveillance procedures. Specifically, in 11 of the case reports included in this review, the diagnosis was made following a screening colonoscopy prompted by positive fecal occult blood test results. In four studies, the diagnosis was reached during oncologic follow-up: in three of these cases, the detection of elevated tumor markers (Cancer Antigen 125 (CA 125), Carcinoembryonic Antigen (CEA) and Cancer Antigen 19.9 (CA 19.9)) and subsequent confirmation by PET-CT or CT scan. Two studies (3.1%) do not report symptoms referred by patients at time of diagnosis. The median survival after diagnosis of the metastatic disease, expressed in months, was 12 (IQR = 27.5). Eleven studies reported death of patients after a median of 12 months (IQR = 20 months). Two studies reported patient death; however, they did not provide information regarding the timing of the event in relation to the initial diagnosis. Additionally, 24 studies did not include any follow-up data. As a result, these studies were excluded from the statistical analysis.

Our review shows a heterogeneous therapeutic approach. Systemic treatments such as chemotherapy (including paclitaxel, gemcitabine, and taxane-based regimens) and endocrine therapy (anastrozole, letrozole, tamoxifen, and fulvestrant, often combined with CDK4/6 inhibitors like palbociclib or abemaciclib) were commonly reported as first-line strategies. Surgical intervention was performed in 32 out of 64 cases (50%), primarily for obstructive complications or, less frequently, bleeding and perforation. In other case reports (6% of case reports included), surgery was performed as a preventive measure in patients with progressive disease and high risk of bowel obstruction (SDC4). Table 3 summarizes the therapeutic strategies.

**Table 3:** Therapeutic strategies adopted in case reports included in our review

Author	Treatment	Reason for surgical approach
Takeuchi et al. [7]	Capecitabine	
Jansen van Rensburg et al. [8]	Surgery + adjuvant A.I	Not reported
Highley et al. [9]	Paclitaxel	
Khan et al. [10]	CT	
Noor et al. [11]	Paclitaxel + Gemcitabine	
Bering et al. [12]	Palbociclib + Letrozole	
Abid et al. [13]	Anastrozole	
Takedomi et al. [14]	CT	
Abu Zaanona et al. [15]	Letrozole + Palbociclib	
Inoue et al. [16]	Laparoscopic Sigmoidectomy + Fulvestrant and Abemaciclib	Symptoms of stenosis; preventive surgery
Tsujimura et al. [17]	Open ileocecal resection + Adjuvant Letrozole	Symptoms of stenosis
Feng et al. [18]	Not Reported	
Kobayashi et al. [19]	Hormonal Therapy	
Arif et al. [20]	Palliative Support	
Jia et al. [21]	Tamoxifene	
Kim et al. [22]	Not Reported	
Signorelli et al. [23]	Right Hemicolectomy + Adjuvant Letrozole	Bowel obstruction
Malhotra et al. [24]	Not reported	
Wang et al. [25]	Right Hemicolectomy	Symptoms of stenosis
Algethami et al. [26]	Not reported	
Zhou et al. [27]	CT + Endocrine Therapy	
Andriola et al. [28]	Left Hemicolectomy	Symptoms of stenosis
Motos-Micó et al. [29]	Not Reported	
Abdallah et al. [30]	CT + A.I	
Michalopoulos et al. [31]	Right Hemicolectomy + Adjuvant CT	Symptoms of stenosis
Schellenberg et al. [32]	Palliative Support	
Matsuda et al. [33]	Neo-Adjuvant CT-RT; Proctectomy	Persistent of Symptoms; no efficacy neo-adjuvant therapy (preventive surgery)
Koleilat et al. [32]	Subtotal colectomy; Taxotere + Xeloda	Bowel obstruction
Mistrangelo et al. [34]	Hartmann’s Procedure	Bowel obstruction
Razzetta et al. [36]	CT	
Cervi et al. [37]	Surgery	
Amin et al. [38]	Hartmann’s Procedure + Letrozole	Symptoms of stenosis
Lopez Deogracias et al. [39]	CT	
Law et al. [40]	Surgery	Bowel Obstruction
Samra et al. [41]	Paclixatel + Fulvestrant	
Gizzi et al. [42]	Surgery + Adjuvant CT	Bowel Obstruction
Dhar et al. [43]	Surgery	Colonic perforation
Maekawa et al. [44]	Surgery + CT	Not reported
Koufopoulos et al. [45]	Hormonal Therapy	
Critchley et al. [46]	Docetaxel + Anastrozole; Capecitabine	
Kachi et al. [47]	Surgery	Symptoms of stenosis
Blachman-Braun et al. [48]	Surgery	Bowel Obstruction
Katz et al. [49]	Right Hemicolectomy + Exemestane	Bowel Obstruction
Falco et al. [50]	Right Hemicolectomy	No Information
Imai et al. [51]	Not reported	
Do et al. [52]	Palliative Care	
Schwarz et al. [53]	Not reported	
Ambroggi et al. [54]	1. Surgery, CT + endocrine therapy 2. Epirubicin + Docetaxel; tamoxifene + RT; CT with doxorubicin + Carboplatin	1. Bleeding
Lau et al. [55]	Surgery; Letrozole + RT	Not reported
Santini et al. [56]	Surgery	Bowel Obstruction
Lautliev et al. [57]	Laparoscopic Sigmodectomy; RT + endocrine therapy	Symptoms of stenosis
Abdalla et al. [58]	Letrozole + Denosumab	
Bamias et al. [59]	CT + Surgery; Adjuvant RT and Pamidronate	Symptoms of stenosis
Black et al. [60]	Not reported	
Ng CE et al. [61]	Paclitaxel + stenting	
Ikeda et al. [62]	Laparoscopic Sigmodectomy	Bleeding
Okamura et al. [63]	Right Hemicolectomy	Bowel Obstruction
Nikkar-Esfahani et al. [64]	Hartmann’s Procedure + Letrozole	Symptoms of Stenosis
Osaku et al. [65]	Paclitaxel + Docetaxel + CT	
Rajan et al. [66]	RT + Surgery	Bleeding
Théraux et al. [67]	Surgery + endocrine therapy	Bowel Obstruction
Mostafa et al. [68]	Surgery + CT	Bowel Obstruction
Li et al. [69]	Surgery + Capecitabine	Bleeding
Jones et al. [70]	Right Hemicolectomy + Adjuvant Anastrazole	Not reported

*CT*, chemotherapy; *RT*, radiotherapy; *A.I.*, Aromatase inhibitors

### Quality assessment of the included studies

The risk of bias for included case reports is shown in SDC 5. A total of 64 studies were evaluated for risk of bias across eight methodological domains. One of these, Domain 7, was marked as not applicable (“N-A”) in all cases and was therefore excluded from the analysis. Overall, the studies demonstrated a generally high level of methodological rigor. Domain 1 showed the highest proportion of positive assessments, with 95.3% of studies rated as “Yes.” Similarly, Domain 4 and Domain 2 exhibited strong results, with 89.1% and 87.5% positive assessments, respectively. In contrast, Domain 3 and Domain 6 revealed more limitations. Only 42.2% of studies received a “Yes” rating in Domain 3, while 25.0% were rated as “No” and a notable 32.8% as “Unclear.” Domain 6 presented a similar concern, with only 67.2% of studies rated positively and 32.8% rated as “No.” Importantly, the data for these two domains are likely constrained by the lack of detailed information regarding patient follow-up, which was not consistently or clearly reported across studies. The remaining domains showed relatively favorable distributions. Domain 5 and Domain 8 had “Yes” responses in 82.8% and 85.9% of cases, respectively, suggesting generally good performance in these areas of methodological quality.

## Discussion

Our case is consistent with previously published data: the diagnosis of gastrointestinal metastases occurred approximately seven years after the initial diagnosis of the primary breast tumor. Histological examination of breast primary tumor was an invasive lobular carcinoma, and the immune-histochemical profile of colonic metastasis remained unchanged, showing persistent strong positivity for estrogen and progesterone receptors, in line with the original tumor phenotype.

Gastro-intestinal metastasis from breast cancer is extremely rare: the incidence is estimated around 1% for gastrointestinal disease and only 0.1% for colon cancer [4]. Previous studies regarding the incidence of colorectal metastasis were conducted. A comprehensive review conducted by Wiisanen and Kaur [71] in 2015 analyzed 32 breast cancer patients, treated at Mayo Clinic from 2000 to 2013, who had spread to peritoneum or GI tract. The study showed that the most common histology was lobular carcinoma and the most common site of GI metastases was the peritoneum, with 59% of involvement, followed by stomach (17%), colon (15%) and small bowel (15%). Another retrospective analysis conducted by Ambroggi et al. [54] considered 200 metastatic breast cancer patients and showed that for what concern the gastro-intestinal metastatic localization, the most common site was stomach (60%), with a colonic involvement of only 12%. According to the analysis above and previous literature review, lobular carcinoma is most frequently associated with gastro-intestinal metastasis than ductal carcinoma.

The prognosis is very poor and the main reasons are the long time of tumor recurrences onset and the lack of specific symptoms. In fact, patients with gastro-intestinal metastasis usually have not relevant clinical symptoms: the most common include dyspepsia, nausea and vomiting, weight loss and obstruction in emergency setting. Due to the specificity of the above symptoms, in case of GI lesion, an initial diagnosis of primary gastro-intestinal tumor should be considered, especially if time interval from breast tumor is long.

For this reason, the immune-histochemical evaluation is a crucial aspect for metastatic breast tumor diagnosis. In addition to evaluation of hormone-receptor (ER, PgR and HER2/Neu) expression, Gross Cystic Disease Fluid Protein-15 (GCDFP-15), mammaglobin and GATA3 are relatively specific antibodies of breast, and they can be helpful to determine the origin of metastatic disease. In fact, literature review confirmed that sensitivity and specificity of GCDFP-15 are, respectively, 5–74% and 9–100% [60], while for mammaglobin and GATA 3 they are, in order, as follow: 7–84% and 85–100% (mammaglobin) and 32–100% and 71–93% (GATA 3) [60]. Other possible markers are CK7, CK20: CK7 is expressed in 89–98% of non-specified breast tumor, while CK20 positivity is strongly associated with a no-breast origin. So, a CK7 +/CK20 − phenotype should be considered indicative of tumor originating from breast [18].

The immunophenotypic profile of CK7 +/CK20 −/GATA3 +/mammaglobin + with loss of E-cadherin expression is strongly supportive of a breast origin, particularly in the context of invasive lobular carcinoma (ILC). Conversely, a profile of CK20 + and CDX2 + is more characteristic of colorectal adenocarcinomas. It is important to note that no single immunohistochemical marker is definitive. Diagnostic interpretation should rely on a panel of markers and be integrated with histomorphological features and the clinical context, in order to achieve an accurate diagnosis, especially when dealing with metastatic tumors of unknown origin. In our case, immune-histochemical evaluation of only CK7, CK20, GATA3 and hormone-receptors was performed.

A possible mismatch between the receptors in the primary breast tumor and colonic metastasis is possible. In our review, seven studies (10.9%) reported these receptors mismatched. In this case, three studies (4.7% of case reports included) described an only modification of PgR in the colonic metastasis compared to primary breast tumor, one case (1.6% of case reports included) reported a modification of ER, while a mismatch of both progesterone and estrogenic receptors is described in the remaining three studies (10.9% of case reports included). The significance of hormonal mismatched is not still clear. However, a study conducted by Lindstrom et al. [72] found that progesterone receptor (PgR) status showed the highest rate of discordance at 38.6%, followed by estrogen receptor (ER) at 21%, and HER2/Neu at 10.8%. The study also distinguished between cases with isolated PgR changes and those involving ER or both ER and PgR alterations and showed that women with ER positive primary breast cancer who transformed in ER negative metastatic disease present an increasing risk of death. These findings highlight the clinical importance of reassessing receptor status in metastatic lesions to guide treatment decisions effectively.

Currently, there is not a clearly defined consensus about the treatment of gastro-intestinal metastasis after breast cancer surgery as well as, there is no indication for routine colonoscopy surveillance in asymptomatic breast cancer patients [73, 74]. However, in the presence of gastrointestinal symptoms, endoscopic evaluation plays a critical role in the early diagnosis. While incidental findings during follow-up are rare, colonoscopy remains essential in guiding the management of symptomatic or high-risk patients. Surgical approach can be performed, but it does not improve the overall survival and prognosis of patients, that remain poor: it should be considered only for palliative purpose and in case of life-threatening complications [75, 76]. In our case report, surgical intervention was mandatory because of a progressive intestinal obstruction, unresponsive to the medical therapy attempts. The procedure was considered as a preventive measure to avoid a complete intestinal obstruction. Ultimately, the surgical approach resulted in a marked improvement in the patient’s quality of life. Chemotherapy, radiotherapy, and endocrine therapy are the main treatment and are chosen based on biologic features of disease and previous treatment, such as in breast cancer with metastases in other sites. Our patient was treated with non-steroidal aromatase inhibitors combined to CDK4/6 inhibitors due to presence of hormonal receptors and lack of HER2 overexpression. This combination therapy is today the preferred regimen in this setting. Although the combination of CDK4/6 inhibitors with endocrine therapy is considered the standard first-line treatment for hormone receptor-positive (HR +), HER2/Neu negative metastatic breast cancer, it is important to note that most of the supporting evidence is derived from studies focusing on breast cancer in general, rather than specifically on GI metastases. For instance, a comprehensive meta-analysis encompassing 19,004 patients demonstrated significant improvements in progression-free survival (PFS), overall survival (OS), objective response rate (ORR), disease control rate (DCR), and clinical benefit response (CBR) with the combination therapy in HR +/HER2/Neu negative breast cancer [75]. Therefore, while CDK4/6 inhibitors combined with endocrine therapy represent a preferred treatment strategy, clinical decisions should be individualized, considering prior therapies, comorbidities, and potential toxicity profiles, especially in patients with GI metastases. There are three different CDK4/6 inhibitors approved (Palbociclib, Ribociclib and Abemaciclib) and their toxicity profiles should be considered in treatment’s choice. The most frequent adverse event with Palbociclib and Ribocliclib is neutropenia, while diarrhea is the most common toxicity associated to Abemaciclib. However, an established consensus about the treatment of breast cancer’ GI metastasis is not still available: a tailored therapy should be considered based on patient’s previous medical history and biological features of disease. A conclusive table (Table 4) describes the most relevant results of our review.

**Table 4:** Key results of our study

Outcome	Results of our systematic review
Presenting symptoms	Abdominal pain (32.3%) (*n* = 21); change of bowel habit (24.6%) (*n* = 16); bowel obstruction (9.2%) (*n* = 6); bleeding (7,7%) (*n* = 5); perforation (1.5%) (*n* = 1); routine surveillance procedure (screening colonoscopy or oncologic follow-up) (23%) (*n* = 15)
Biomarkers	ER, PgR, HER2/Neu, CK7, CK20, GATA3
First line of treatment for ER positive, PgR +, HER2/Neu negative tumor	CDK4/6 inhibitors with endocrine therapy
Surgery	Palliative support

*ER*, estrogen receptor; *PgR*, Progesterone Receptor; *HER2/Neu*, Human Epidermal Growth Factor Receptor 2; *GATA3*, GATA Binding Protein 3; *CK7*, Cytokeratin 7; *CK20*, Cytokeratin 20

### Limitations

This review is based solely on case reports and case series, which are subject to inherent methodological limitations, including a high risk of publication bias and limited representativeness of the full clinical spectrum. Heterogeneity and incomplete reporting of key variables, such as staging, follow-up, immunohistochemistry, and treatment rationale, preclude meaningful comparison across cases and prevent statistical pooling or meta-analysis. Follow-up data were missing in a substantial proportion of studies (*n* = 25), limiting the assessment of time-to-event outcomes and introducing potential selection bias and reduced statistical power. Risk-of-bias assessment confirmed frequent deficiencies in follow-up reporting, which was adequate in only 67.2% of cases. Despite these limitations, case-based evidence remains valuable for rare conditions. This review summarizes available clinical patterns and management approaches, providing a preliminary framework to inform future research and support the development of standardized data collection and clinical guidelines.

## Conclusion

Colonic metastasis from breast cancer is a rare, but a possible eventuality. In the clinical follow-up of breast cancer patients, endoscopic assessment could be considered when gastrointestinal symptoms arise or in case of suspicious findings, although current evidence does not support routine surveillance. The long-time interval from the primary breast tumor and the colonic metastasis onset, as well as the aspecific clinical symptoms make the diagnosis difficult and easily missed: rare sites of metastases and the different receptor expression must be considered in order to obtain a correct and prompt diagnosis. Surgery is typically reserved for palliation of obstruction, bleeding, or perforation; survival benefit has not been demonstrated in comparative studies.

## Supplementary Information

Below is the link to the electronic supplementary material.

ESM 1 (DOCX 24.9 KB)

ESM 2 (DOCX 14.6 KB)

ESM 3 (DOCX 88.2 KB)

ESM 4 (DOCX 209 KB)

ESM 5 (DOCX 20.0 KB)

## Data Availability

The data used to support the finding of this study are included within the article.
