# Supplementary material for: Colonic metastasis from breast carcinoma: A case report and systematic review of a rare clinical scenario
Source: Int J Colorectal Dis. 2026 Feb 7;41(1):61. doi: 10.1007/s00384-026-05102-0 (PMC12881134; doi:10.1007/s00384-026-05102-0)
Supplement: Supplementary file 1 — (DOCX 24.9 KB) [file 384_2026_5102_MOESM1_ESM.docx]

| **Abbreviation** | **Definition** |
| --- | --- |
| ER | Estrogen Receptor |
| PgR | Progesterone Receptor |
| HER2/Neu | Human Epidermal Growth Factor Receptor 2 |
| ILC | Invasive Lobular Carcinoma |
| LC | Lobular Carcinoma |
| IDC | Invasive Ductal Carcinoma |
| DC | Ductal Carcinoma |
| CK7 | Cytokeratin 7 |
| CK20 | Cytokeratin 20 |
| CDX2 | Caudal-Type Homeobox Transcription Factor 2 |
| PFS | Progression free survival |
| OS | Overall survival |
| AI | Aromatase Inhibitor |
| CT | Chemotherapy |
| RT | Radiotherapy |
| FU | Follow-up |
| NED | No Evidence of Disease |
| GCDFP-15 | Gross Cystic Disease Fluid Protein-15 |

**SDC 1.** Abbreviations and their definitions as used in the manuscript.
