# Supplementary material for: Colonic metastasis from breast carcinoma: A case report and systematic review of a rare clinical scenario
Source: Int J Colorectal Dis. 2026 Feb 7;41(1):61. doi: 10.1007/s00384-026-05102-0 (PMC12881134; doi:10.1007/s00384-026-05102-0)
Supplement: Supplementary file 4 — (DOCX 209 KB) [file 384_2026_5102_MOESM4_ESM.docx]

**SDC 4.** The figure provides a graphical representation of the reasons for surgical treatment.
