# Supplementary material for: Colonic metastasis from breast carcinoma: A case report and systematic review of a rare clinical scenario
Source: Int J Colorectal Dis. 2026 Feb 7;41(1):61. doi: 10.1007/s00384-026-05102-0 (PMC12881134; doi:10.1007/s00384-026-05102-0)
Supplement: Supplementary file 5 — (DOCX 20.0 KB) [file 384_2026_5102_MOESM5_ESM.docx]

| **Author** | **Domain 1** | **Domain 2** | **Domain 3** | **Domain 4** | **Domain 5** | **Domain 6** | **Domain 7** | **Domain 8** |
| --- | --- | --- | --- | --- | --- | --- | --- | --- |
| Tekeuchi [5] | Yes | Yes | Yes | Yes | Yes | Yes | N-A | Yes |
| Jansesn van Rensburg [6] | Yes | Yes | Yes | Yes | Yes | Yes | N-A | Yes |
| Khan [8] | Yes | Yes | Yes | No | No | Yes | N-A | No |
| Higley [7] | Yes | Yes | Yes | No | Yes | Yes | N-A | Yes |
| Noor [9] | Yes | Yes | Yes | Yes | Yes | Yes | N-A | Yes |
| Bering [10] | Yes | Yes | Unclear | Yes | Yes | No | N-A | Yes |
| Abid [11] | Yes | Yes | Unclear | Yes | No | No | N-A | Unclear |
| Takedomi [12] | Yes | Yes | No | No | Yes | No | N-A | No |
| Abu Zaanona [13] | No | No | Yes | Yes | Yes | Yes | N-A | Yes |
| Inoue [14] | No | Yes | Yes | Yes | Yes | Yes | N-A | Yes |
| Amin [36] | Yes | Yes | No | Yes | Yes | No | N-A | Yes |
| Mostafa [66] | Yes | Yes | No | Yes | Yes | No | N-A | Yes |
| Tsujimura [15] | Yes | Yes | Unclear | Yes | No | Yes | N-A | Yes |
| Feng [16] | Yes | Yes | No | Yes | No | No | N-A | No |
| Kobayashi [17] | Yes | Yes | Yes | Yes | Yes | Yes | N-A | Yes |
| Théraux [65] | Yes | Yes | Unclear | Yes | Yes | Yes | N-A | Yes |
| Malhotra [22] | Yes | No | No | No | No | No | N-A | No |
| Wang [23] | Yes | Yes | Unclear | Yes | Yes | Yes | N-A | Yes |
| Algethami [24] | Yes | Yes | No | Yes | Yes | No | N-A | Yes |
| Zhou [25] | Yes | Yes | Unclear | Yes | Yes | Yes | N-A | Yes |
| Andriola [26] | Yes | No | No | Yes | Yes | No | N-A | Yes |
| Motos-Mico [27] | Yes | No | No | Yes | Yes | No | N-A | Yes |
| Abdallah [28] | Yes | Yes | Unclear | Yes | No | Yes | N-A | Unclear |
| Michalopoulos [29] | Yes | Yes | Yes | No | Yes | Yes | N-A | Yes |
| Michalopoulos [29] | Yes | Yes | No | No | Yes | No | N-A | Unclear |
| Schellenberg [30] | Yes | Yes | Yes | Yes | Yes | Yes | N-A | Yes |
| Matsuda [31] | Yes | Yes | No | Yes | Yes | No | N-A | Yes |
| Koleilat [32] | Yes | Yes | Unclear | Yes | Yes | Yes | N-A | Yes |
| Mistrangelo [33] | Yes | Yes | Yes | No | Yes | Yes | N-A | Yes |
| Razzetta [34] | Yes | Yes | No | Yes | No | No | N-A | No |
| Cervi [35] | Yes | Yes | No | Yes | Yes | No | N-A | Yes |
| Lopez Deogracias [37] | Yes | Yes | Yes | Yes | Yes | Yes | N-A | Yes |
| Law [38] | Yes | Yes | No | Yes | Yes | No | N-A | Yes |
| Samra [39] | Yes | No | Yes | No | No | Yes | N-A | No |
| Kim [20] | Yes | Yes | No | Yes | Yes | No | N-A | Yes |
| Signorelli [21] | Yes | Yes | Unclear | Yes | Yes | Yes | N-A | Yes |
| Gizzi [40] | Yes | Yes | Unclear | Yes | Yes | Yes | N-A | Yes |
| Dhar[41] | Yes | Yes | No | Yes | Yes | No | N-A | Yes |
| Maekawa [42] | Yes | Yes | Yes | No | Yes | Yes | N-A | Yes |
| Koufopoulos [43] | Yes | No | No | Yes | No | No | N-A | No |
| Critchley [44] | Yes | Yes | Unclear | Yes | Yes | Yes | N-A | Yes |
| Kachi [45] | Yes | Yes | Unclear | Yes | Yes | Yes | N-A | Yes |
| Blachman-Braun [46] | Yes | Yes | No | Yes | Yes | No | N-A | Yes |
| Jia [19] | Yes | Yes | Unclear | Yes | Yes | Yes | N-A | Yes |
| Arif [18] | Yes | No | Yes | Yes | No | Yes | N-A | No |
| Katz [47] | Yes | No | No | Yes | Yes | No | N-A | No |
| Falco [48] | Yes | Yes | Unclear | Yes | Yes | Yes | N-A | Yes |
| Do [50] | Yes | Yes | No | Yes | Yes | No | N-A | Yes |
| Jones [68] | Yes | No | Unclear | Yes | Yes | Yes | N-A | Yes |
| Imai [49] | Yes | Yes | No | Yes | Yes | No | N-A | Yes |
| Nikkar-Esfahani [62] | Yes | Yes | Unclear | Yes | Yes | Yes | N-A | Yes |
| Ikeda [60] | No | No | Unclear | No | Yes | Yes | N-A | No |
| Okamura [61] | No | No | Unclear | Yes | Yes | Yes | N-A | No |
| Schwarz [51] | Yes | Yes | Unclear | No | Yes | Yes | N-A | Yes |
| Ambroggi [52] | Yes | Yes | Yes | Yes | Yes | Yes | N-A | Yes |
| Lau [53] | Yes | Yes | Unclear | Yes | Yes | Yes | N-A | Yes |
| Santini [54] | Yes | Yes | Unclear | Yes | Yes | Yes | N-A | Yes |
| Laoutliev [55] | Yes | No | No | Yes | Yes | No | N-A | No |
| Abdalla [56] | Yes | Yes | No | Yes | Yes | No | N-A | Yes |
| Bamias [57] | Yes | Yes | Yes | Yes | Yes | Yes | N-A | Yes |
| Black [58] | Yes | Yes | No | Yes | No | No | N-A | No |
| NgCE [59] | Yes | Yes | Unclear | Yes | Yes | Yes | N-A | Yes |
| Osaku [63] | Yes | Yes | Yes | No | Yes | Yes | N-A | Yes |
| Rajan [64] | Yes | Yes | Unclear | Yes | Yes | Yes | N-A | Yes |
| Li [67] | Yes | Yes | Unclear | Yes | Yes | Yes | N-A | Yes |

**SDC 1.** Quality Assessment of case reports included using the JBI Critical Appraisal Checklist for case reports. Domain 1: were patient’s demographic characteristics clearly described?; Domain 2: was the patient’s history clearly described and presented as a timeline?; Domain 3: was the current clinical condition of patients on presentation clearly described?; Domain 4: were diagnostic tests or assessment methods and the results clearly described; Domain 5: was the intervention (s) or treatment procedure (s) clearly described?; Domain 6: was the post-intervention clinical condition clearly described?; Domain 7: were adverse events (harms) or unanticipated event identified and described?; Domain 8: does the case report provide takeaway lessons?
